# In vivo phage display identifies novel peptides for cardiac targeting

**DOI:** 10.1038/s41598-024-62953-9

**Published:** 2024-05-28

**Authors:** Alena Ivanova, Franziska Kohl, Hernán González-King Garibotti, Renata Chalupska, Aleksander Cvjetkovic, Mike Firth, Karin Jennbacken, Sofia Martinsson, Andreia M. Silva, Ida Viken, Qing-Dong Wang, John Wiseman, Niek Dekker

**Affiliations:** 1 Discovery Biology, Discovery Sciences, BioPharmaceuticals R&D, AstraZeneca, Pepparedsleden 1, Mölndal, 431 50 Gothenburg, Sweden; 2 Translational Genomics, Discovery Sciences, BioPharmaceuticals R&D, AstraZeneca, Pepparedsleden 1, Mölndal, 431 50 Gothenburg, Sweden; 3 Department of Medical Biochemistry and Biophysics, Karolinska Institutet, Solnavägen 1, Solna, 171 77 Stockholm, Sweden; 4 Bioscience Cardiovascular, Research and Early Development, Cardiovascular, Renal and Metabolism (CVRM), BioPharmaceuticals R&D, AstraZeneca, Pepparedsleden 1, Mölndal, 431 50 Gothenburg, Sweden; 5 Data Sciences and Quantitative Biology, Discovery Sciences, BioPharmaceuticals R&D, AstraZeneca, Cambridge, CB2 0AA UK

**Keywords:** Bioinformatics, Cardiovascular biology, Peptide delivery

## Abstract

Heart failure remains a leading cause of mortality. Therapeutic intervention for heart failure would benefit from targeted delivery to the damaged heart tissue. Here, we applied in vivo peptide phage display coupled with high-throughput Next-Generation Sequencing (NGS) and identified peptides specifically targeting damaged cardiac tissue. We established a bioinformatics pipeline for the identification of cardiac targeting peptides. Hit peptides demonstrated preferential uptake by human induced pluripotent stem cell (iPSC)-derived cardiomyocytes and immortalized mouse HL1 cardiomyocytes, without substantial uptake in human liver HepG2 cells. These novel peptides hold promise for use in targeted drug delivery and regenerative strategies and open new avenues in cardiovascular research and clinical practice.

## Introduction

Heart failure (HF), remains a leading cause of mortality worldwide^1^. Myocardial infarction (MI) is a common cause of heart failure and results in substantial loss in cardiomyocyte numbers and ischemia in the heart^2^. Despite significant progress in cardiovascular research, the development of targeted therapies for the damaged heart poses substantial challenges^3–5^.

Highly selective and tightly regulated vasculature of the heart creates obstacles for therapeutics, both chemicals and biologicals, to penetrate and reach the cardiac tissue^6^. Additionally, heart’s efficient clearance mechanisms, including rapid blood flow and lymphatic drainage, can reduce the concentration and duration of therapeutic exposure^7^. Inflammatory processes associated with cardiac diseases can lead to increased vascular permeability^8^. Animal models of ischemia–reperfusion allow for exploiting the complex pathophysiological environment occurring in the heart after reperfusion^9^. These models enable the identification of cardiac targeting molecules, potentially offering new avenues for targeted drug delivery and regenerative strategies.

The phage display technology enables the presentation of a diverse protein and peptide repertoire on the surface of bacteriophages, enabling identification of peptide sequences that bind to disease-specific targets in vivo^10,11^. Phage display libraries exposure against cardiac tissue samples obtained from post-MI animal models introduced peptides that exhibit affinity to cardiac tissues affected by MI^12^. Although there is a high demand for peptides targeting damaged myocardium, the number of such peptides currently available for research application is limited.

Traditionally, the analysis of phage display experimental results involves sequencing a limited number of individual phage clones to identify candidate peptides^13–16^. This approach leads to loss of information and restricts the diversity of discovered target-binding peptide populations. The application of Next-Generation Sequencing (NGS), a high-throughput approach, has revolutionized the analysis of phage display libraries^17–21^. By sequencing the DNA insert of phages obtained from a phage display screen, it is possible to get information about the frequency of all peptide sequences in the sample, enabling the identification of highly abundant or rare peptides. However, the analysis of NGS phage display data lacks convenient and standard methods, making it challenging to establish a consistent framework. A universal and unbiased approach is needed to ensure comparability across studies and experimental settings. Furthermore, high sequence similarity among identified peptides presents a hurdle that requires specialized analysis techniques not covered by existing approaches. In cases where phage display targets a specific tissue region with low phage recovery, other challenges arise. The limited number of recovered phages can result in reduced sequence diversity, potentially affecting peptides’ representation in the assay and requiring special approaches for data analysis. Thereafter, novel approaches that integrate bioinformatic tools are required for analysing phage display data.

In this study, we investigate the potential of phage display technology and NGS in advancing the discovery of peptides targeting ischemic heart region. We employ PhD-12 and TriCo-20 phage display libraries with wide ranges of peptide sequences, aiming to identify novel peptides in vivo. We get over 270,000 unique NGS reads from the target organs, with over 90,000 different peptides. We compared established data analysis approaches with newly developed pipelines to identify ischemic heart-specific peptides from this cohort. Top 20 hits we validated in vitro and identified 3 peptides that confirmed specific uptake by human induced pluripotent stem cell (iPSC)-derived cardiomyocytes and immortalized mouse cardiomyocytes HL1, two of them demonstrated uptake by hypoxia and ischemia-stimulated iPSC-derived cardiomyocytes.

## Results

### In vivo phage display to identify peptides binding to the ischemic cardiac region

**Figure 1:**
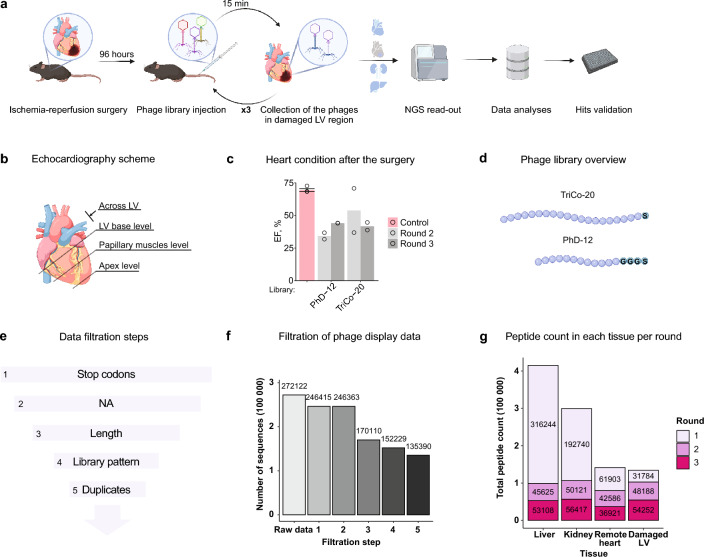
Identification of peptides targeting ischemic cardiac regions using phage display. (**a**) Illustration of the experimental workflow. Ninety-six hours after ischemia–reperfusion injury was induced, mice were injected with phage display libraries via the tail vein for in vivo phage display. Animals were terminated 15 min later and their damaged left ventricular (LV) region, remote heart, kidney, and liver were collected. Phages from the damaged LV tissue were amplified and injected into new animals with ischemia–reperfusion surgery for the next round of phage display. After the third round of phage display, phages from liver, kidney, healthy heart, and scar tissues were amplified and sequenced. Data analysis was followed by peptide validation. (**b**) To evaluate heart condition after ischemia–reperfusion surgery, echocardiography was performed across LV long-axis; at the level of the ventricle base; at approximately the level of the papillary muscles; and at the apex level. (**c**) Ejection fraction (EF) of the animals at day 3 after ischemia–reperfusion injury. Ejection fraction of healthy heart is indicated by a pink bar. N = 2 for groups treated with the phages and N = 4 for the healthy control. (**d**) Phage libraries overview. TriCo-20 includes peptides of 20 amino acids attached through a serine linker to the phage coat protein. PhD-12 consists of 12 amino acids peptides with a glycine-serine linker. (**e**) Schematics for data filtration steps. First, reads with stop codons in the peptide sequences were eliminated. Next, reads that were not translating to amino acid sequences were removed from the initial dataset. Peptide length and library patterns allowed us to sort out unrelated reads. Last, duplicated amino acid sequences were merged. (**f**) Total number of peptides at each filtration step as described in (**a**). (**g**) Filtered data set was used to calculate the number of reads in each tissue per panning round of the phage display experiment.

Phage display technology can be employed to identify targeting peptides. We performed selection of the phages in a mouse model of ischemia–reperfusion (Fig. 1A). The ischemia–reperfusion model used involved temporary restriction of blood supply to the heart followed by its restoration and mimicked the clinical situation in patients with MI^9^. We conducted our experiments four days after the surgery to increase the chances of identifying peptides that specifically recognized the diseased heart tissue. To evaluate heart function after the surgery, echocardiography was performed and the ejection fraction (EF) was reduced to less than 50% for all examined mice except one, which had EF at the level of a healthy heart (70–75%) (Fig. 1B,C).

With the aim for identifying peptide sequences specifically associated with myocardial infarction, we subjected phage display libraries to three rounds of selection against ischemic left ventricular (LV) cardiac tissue. We used two commercial phage display libraries, TriCo-20 and PhD-12, that displayed random peptide sequences in the pIII coat protein of M13 bacteriophage (Fig. 1D). These libraries were administered to mice after ischemia–reperfusion by intravenous tail vein injection (Fig. 1A). These phages were allowed to circulate within the bloodstream to encourage interaction with various cell types, including cardiomyocytes in the damaged heart region, for 15 min then were washed away by perfusing animals before termination. Damaged LV cardiac tissue was isolated and retained phages were recovered and amplified for the next round of screen. We additionally collected remote heart tissue (the rest of the heart after removing damaged LV and without aorta), liver and kidney as controls for binding specificity. After the last round of screen, phages from tissues of interest were collected and the DNA sequences encoding the displayed peptides were determined by NGS.

### Sequential rounds of phage display increase the proportion of peptides in the heart over other tissues

The NGS data obtained from the phage display experiments underwent a series of filtration steps to remove artifacts, noise in order to improve the quality of the data (Fig. 1E). Sequences containing stop codons or non-amino acid reads were eliminated first, the remaining sequences were then checked for conformity to the library length and pattern to ensure the presence of the correct amino acids preceding the targeting peptides. Finally, any duplicated peptide sequences originated from distinct DNA sequences were merged, ensuring a unique representation of the identified peptides in the final dataset (Fig. 1F).

The total peptide count changed over the rounds of phage display selection. The total peptide count was high in the first round (322761 for PhD-12, 279910 for TriCo-20), reflecting the diversity of peptides presented in the starting phage library (Fig. 1G, Supplementary Fig. S1A). As the selection progressed, peptides that did not bind specifically to the target of interest were gradually depleted from the population, leading to reduced total peptide count of approximately threefold (104660 for PhD-12, 96038 for TriCo-20). Conversely, the total peptide count of the phage population from the previously ischemic LV region increased by 41% due to the amplification of phages displaying peptides with higher affinity for the target (Fig. 1G). Both libraries showed a comparable total number of peptides in different tissues (Supplementary Fig. S1A,B), while in the last round over three times more phages were detected in the library PhD-12 in the remote heart region (Supplementary Fig. S1C).

### Bioinformatic approaches to identify cardiac targeting peptides in the phage display data set

**Figure 2:**
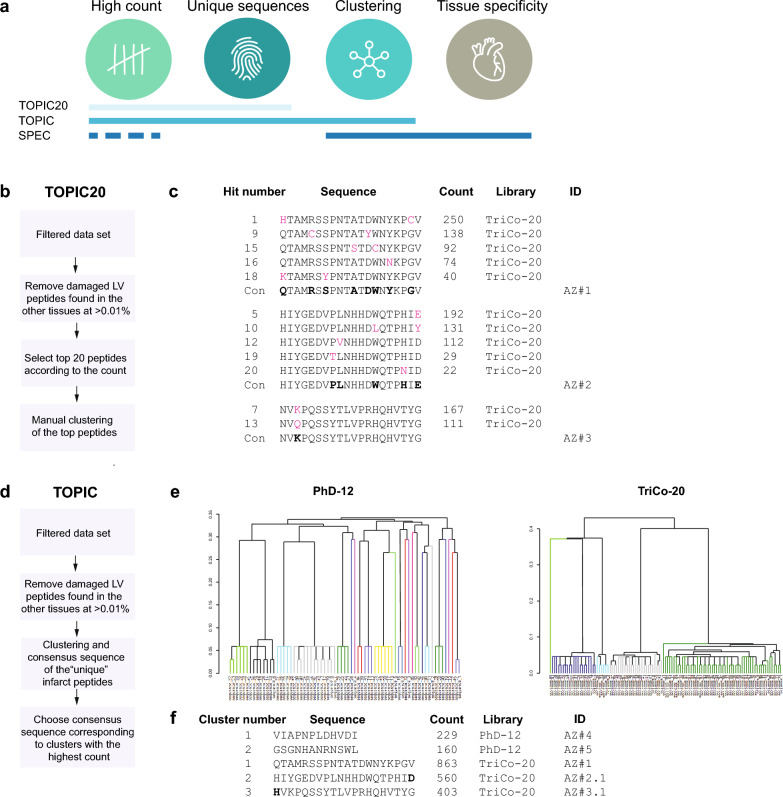
Bioinformatic analyses of the phage display data to identify hits. (**a**) Illustration of bioinformatic approaches for analyzing the in vivo phage display data. The key parameters included in the analysis are high count, unique sequences, clustering, and tissue specificity. (**b**) Description of the TOPIC20 data analysis approach, which combines filtering and manual clustering of the top 20 peptides. (**c**) Consensus sequence analysis of the top 20 hits identified in TOPIC20. (**d**) Description of the TOPIC data analysis approach, which includes filtering and clustering of all filtered peptides. (**e**) Overview of the clustering of the peptide sequences after the filtering step in TOPIC. (**f**) Consensus sequences corresponding to top clusters identified as hits in TOPIC. Amino acids differences between consensus sequences identified by TOPIC20 and TOPIC (peptides AZ#2 vs. AZ#2.1, AZ#3 vs. AZ#3.1) are highlighted in bold. Count assigned to the consensus sequence is a sum of peptide counts in the cluster.

For further data analysis, we applied three complementary approaches Tissue-Oriented Peptide Identification and Clustering of the top 20 hits (TOPIC20), Tissue-Oriented Peptide Identification and Clustering (TOPIC) and Specificity Evaluation by Clustering (SPEC) to identify cardiac targeting peptides (CTPs) within the obtained dataset (Fig. 2A). TOPIC and TOPIC20 are more traditional approaches, that considered peptide count and uniqueness of sequences for the ischemic LV region, while SPEC is more advanced approach that additionally employed clustering techniques to identify potential similarities between peptides and investigated their tissue specificity.

#### Tissue-oriented peptide identification and clustering of the top 20 hits (TOPIC20): tissue-specific filtering and manual clustering of the top hits

To identify potential CTPs, we initially considered the unique peptide count, which provided an indication of the abundance of peptides in a tissue compared to others. In this approach, we evaluated the peptides that were found in the ischemic LV region starting from round 3 (Fig. 2B). Then, we examined if these peptides were also present in other tissues with a counts greater than 0.01% of total counts in the tissue in the round 3, this threshold allowed us to account for rare events of nonspecific binding. If a peptide was detected in other tissues above this threshold, it was considered non-specific to the previously ischemic LV tissue and was removed from the dataset.

To further analyse the remaining peptides, we selected the top 20 hits based on their counts. These selected peptides were then subjected to manual clustering based on sequence similarity to identify consensus sequences (Fig. 2C) and three distinct clusters were identified. To generate a consensus sequence from each identified peptide cluster, we assigned each amino acid in the cluster a “weight” corresponding to the count of the peptide it belonged to. This weighting scheme allowed us to prioritize amino acids that appeared more frequently, indicating their higher representation and potential importance, within the cluster. Subsequently, for each position within a peptide sequence, the amino acid with the highest count in that cluster was selected as the representative for that position in the consensus sequence. Based on this approach, we identified three consensus peptides from the TriCo-20 library as hits (Fig. 2C), but no hits with comparable counts were found in the PhD-12 library.

#### Tissue-oriented peptide identification and clustering (TOPIC): tissue specific filtering and Clustering of the filtered peptides

TOPIC20 approach has an issue of potentially losing relevant peptides by focusing solely on the top twenty hits. To mitigate this, we employed a different strategy that involved clustering all the peptides after applying the filtering step (Fig. 2D). After applying the same filtration strategy of removing peptides that were also present in other tissues at a frequency greater than 0.01% as in the previous approach, we clustered all the remaining peptides together as described in “Materials and methods”. This approach allowed us to capture a broader range of potentially relevant peptides, including those that had lower abundance but still exhibited important sequence similarities. Following the clustering step, we examined the clusters with most counts, which represented groups of peptides that shared common features or conserved regions (Fig. 2E). From these clusters, we could identify consensus sequences, which were representative sequences that summarized the shared characteristics of the peptides within each cluster. Count assigned to consensus sequence was a sum of peptide counts in the cluster.

We took the consensus sequence from the clusters with the highest count. Three hits belonged to the TriCo-20 library and were identical to hits identified in the previous approach, apart from a single amino acid replacement at the terminus (C-terminal E → D for AZ#2.1 and N-terminal N → H for AZ#3.1) (Fig. 2F). In addition, two hits were identified from the PhD-12 library (Fig. 2F). We did not identify these hits in the PhD-12 library using the previous approach because all peptide candidates had low counts, which became high once we clustered similar peptides together and assigned sum of the peptide counts to the consensus sequence.

#### Specificity evaluation by clustering (SPEC): clustering of all peptides and tissue specificity of the clusters

**Figure 3:**
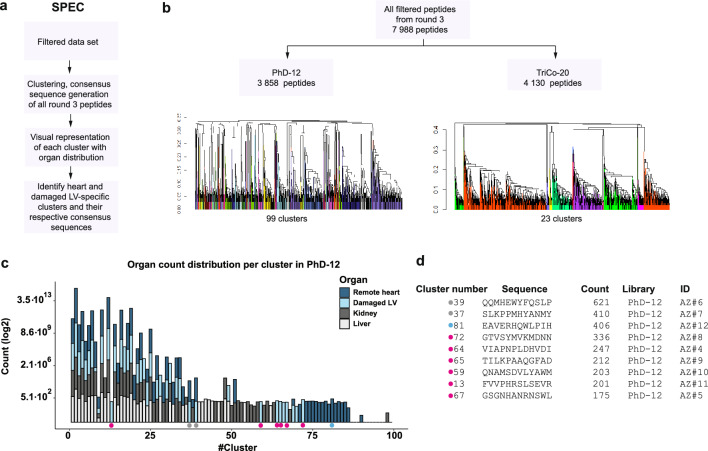
Implementation of global clustering to bioinformatic analyses of the phage display data. (**a**) Description of the SPEC data analysis approach. All peptides from panning round 3 were clustered and the clusters’ tissue representation was examined afterward. (**b**) Clustering of all peptides identified in panning round 3 from libraries PhD-12 and TriCo-20. (**c**) Visual representation of identified clusters across different tissues in library PhD-12. The sum of the count of each peptide from each cluster is represented on the y-axis, cluster number on the x. Magenta circles indicate damaged LV area-specific clusters with the top 6 counts. The grey circles indicate clusters with specificity to the damaged LV region and the remote heart. The blue circle indicates the best hit among remote heart-specific clusters. (**d**) Consensus sequence of the clusters marked in (**c**). Count assigned to the consensus sequence is a sum of peptide counts in the cluster.

In TOPIC and TOPIC20 approaches we used pre-defined threshold to address peptide tissue specificity. In order to ensure that potentially important peptides were not overlooked applying this selection criteria we developed an alternative comprehensive approach that does not relying on this selection criteria (Fig. 3A). We first clustered all 7 988 uniquely identified peptides (Fig. 3B), we then focused on allocating these clusters within specific tissues, including the damaged LV region, remote heart, kidney and liver. We investigated each cluster representation across the tissues (Fig. 3C, Supplementary Fig. S2A). We prioritized clusters that were exclusive to the previously ischemic LV and those that showed a high number of reads in the whole heart (damaged LV + remote heart) for further investigation and downstream experiments (Fig. 3D). With this approach, we identified 8 hits, all of which were from the PhD-12 library. None of the clusters identified in the TriCo-20 library were specific for the damaged LV or for the remote heart (Supplementary Fig. S2A). There were a lot of clusters identified in the remote heart in library PhD-12 (Fig. 3C), we therefore additionally included an extra cluster from this area to the in vitro validation.

We also assessed whether it is necessary to consider clusters behaviour across all three rounds of the phage display experiment to confidently identify hits (Supplementary Fig. S2B). Thus, clusters specific for the damaged heart regions should get more counts in this region and less counts in the other tissues over the rounds. To explore this, we first clustered all the peptides identified in all the rounds of the phage display. Next, we visualized the presentation of clusters in the damaged myocardium and remote heart, the kidney and the liver over the course of three rounds (Supplementary Fig. S2C,D), which allowed us to identify clusters that were enriched specific to the target tissue. From the previously identified hits, three from the PhD-12 library showed enrichment in the damaged myocardium and remote heart, while number of counts reduced in the kidney and liver over the rounds (Supplementary Fig. S2E). This way of analysing the data appeared to be quite stringent, as only a few candidates from previously identified clusters satisfied the enrichment criteria.

Collectively, these approaches allowed us to comprehensively analyze the data obtained from different tissues, focusing on the identification of CTPs specific to the ischemic heart region. By considering factors such as peptide count, uniqueness, clustering, tissue specificity, and enrichment patterns, we uncovered novel potential CTPs.

### In vitro validation of identified CTPs

After identifying hits through various bioinformatic analyses of the data, our next objective was to validate the results in in vitro experiments. To allow the visualisation of the peptides, we synthetised selected peptides AZ#1–AZ#12 with an additional lysine residue at the C-terminus to serve as the linker to the FITC fluorophore. We used human liver cells HepG2 as a negative control to assess non-specific peptide binding and used human induced pluripotent stem cell (iPSC)-derived cardiomyocytes and immortalized mouse cardiomyocytes HL1 to evaluate the targeting potential of the peptides toward cardiac cells. Human induced pluripotent stem cell (iPSC)-derived cardiomyocytes provided us with a relevant model of human cardiomyocytes. We confirmed the cardiomyocyte state of the iPSC-derived cardiomyocytes by immunofluorescence analysis of cardiac troponin T (Supplementary Fig. S3A). In order not to miss hits that are specific for mouse cardiomyocytes we also used immortalized mouse cardiomyocytes HL1.

We incubated cells with 10 μM of the selected peptides and Myc peptide as a non-targeting control for one hour, removing the peptides by replacing the media with imaging media, which also facilitated clear visualization of the cells, and acquired images using a high-throughput confocal microscope.

**Figure 4:**
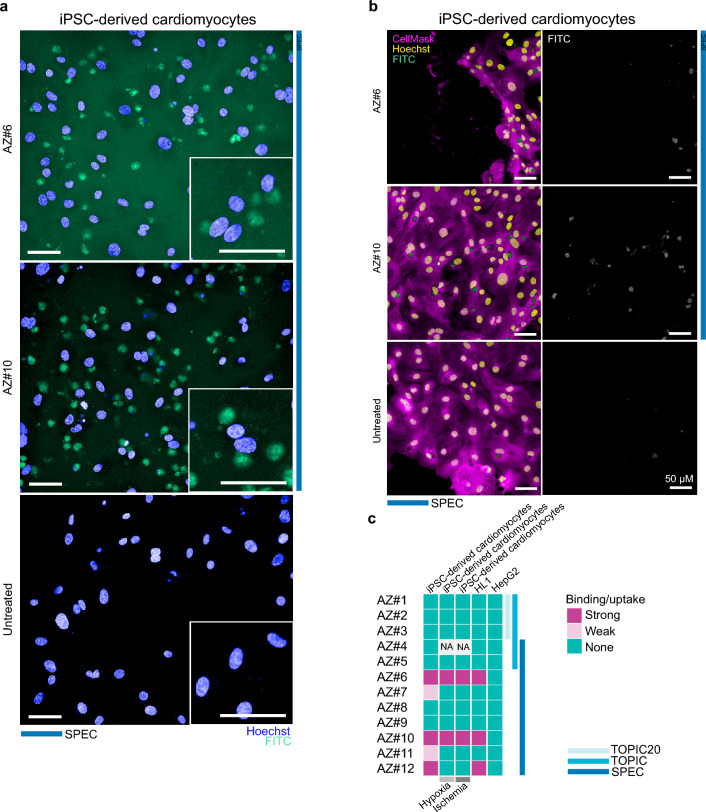
In vitro validation of identified peptides with human iPSC-derived cardiomyocytes. (**a**) Representative confocal fluorescence microscopy images of live human iPSC-derived cardiomyocytes incubated for 1 h with indicated peptides labeled with the FITC fluorophore. Hoechst33342 dye was used for nuclei visualization. Blue lines indicate approaches used to identify the indicated peptide as a hit. Scale bar = 50 μm. (**b**) Human iPSC-derived cardiomyocytes were fixed after 1 h incubation with FITC-labelled peptides. Cell mask and Hoechst33342 dyes were used for membrane and nuclei visualization, respectively. Scale bar = 50 μm. (**c**) Graphical summary of the peptide evaluation. Blue lines at the right indicate approaches where hits were found. Magenta—positive hits, pink—hits with weaker interaction with the cells, green – hits with no binding/uptake detected in the in vitro assay. Grey and light grey lines mark the conditions where cardiomyocytes were hypoxia or ischemia-stimulated. Peptide AZ#4 was not evaluated in hypoxia and ischemia assays as indicated as NA.

We first assessed the binding of the peptides to HL1 cells, which served as a model system closely resembling in vivo conditions. Peptides AZ#6, AZ#10 and AZ#12 gave the strongest fluorescent intensity signals (Supplementary Fig. S3B). The untargeted control peptide Myc bound to the plates, leading to high background signal, which was also observed at similar levels for peptides AZ#2, AZ#3, AZ#4, AZ#9, and AZ#11. Importantly, none of the tested peptides exhibited binding to liver cells above the background level, demonstrating the successful selection against liver binding in our assay (Supplementary Fig. S3C). The staining pattern we observed with the best hits suggested that 1 h incubation is sufficient to detect peptide internalization and intracellular accumulation. Subsequently, to provide insights into the potential translatability of the results obtained from the mouse model to humans, we employed human iPSC-derived cardiomyocytes to further evaluate the targeting properties of the peptides. In line with previous results, peptides AZ#6, AZ#10 and AZ#12 gave the strongest fluorescent intensity signals (Fig. 4A,B, Supplementary Fig. S4A). Peptides AZ#7 and AZ#11 showed weaker staining, suggesting a relatively low affinity for the cells (Supplementary Fig. S4A).

We observed different background signals for different peptides. In order to address if these differences are coming from the unspecific binding of the peptides to the fibronectin coating of the plates, we tested peptide binding to coated and uncoated plates (Supplementary Fig. S4B,C). Indeed, peptides AZ#6, AZ#7, AZ#11, AZ#12 and Myc bind to fibronectin to a higher extent than the others, while once applied to the uncoated plates the pattern changes and Myc and AZ#9 give the most background signal.

After evaluating peptide specificity towards cardiomyocytes over the liver cell model we aimed to check peptide's ability to interact with the “damaged” cardiomyocytes. We incubated cells in a low oxygen environment that would more closely mimic the changes in gene expression networks following a myocardial infarction. Thus, we kept iPSC-derived cardiomyocytes for 24 h in the hypoxia condition (0.8% oxygen) and followed with 6 h of reoxygenation. Afterward, peptides were added, and cells were imaged as previously. Peptide AZ#4 was not included in the validation, since we discovered its tendency to aggregate during the storage which makes it difficult for any future applications from the practical point of view. In line with previous results, peptides AZ#6, and AZ#10 gave the strongest fluorescent intensity signals (Supplementary Fig. S5A–C). In contrast, peptide AZ#12 did not demonstrate affinity to cardiomyocytes in the post-hypoxia condition.

Next, we aimed to evaluate peptides in the in vitro model that even more closely resembles in vivo MI conditions. We additionally changed cell media to nutrition-depleted as recommended in Hakli et al.^22^ during 24 h incubation with low oxygen to mimic the ischemic condition. Afterward cells were returned to maintenance media during 6 h of reoxygenation and treated with peptides as described before. Same as in the hypoxia stimulation experiment peptides AZ#6, AZ#10 gave the strongest fluorescent intensity signals, AZ#12 did not demonstrate affinity to cardiomyocytes (Supplementary Fig. S6A,B).

Altogether it suggests that AZ#6, AZ#10 keep their binding properties to iPSC-derived cardiomyocytes in three in vitro models: with “healthy” cardiomyocytes, with hypoxia-stimulated cardiomyocytes, and with ischemia-stimulated cardiomyocytes. We summarised these results in Fig. 4C.

Notably, all peptides that demonstrated binding to cardiomyocytes were identified using SPEC, emphasizing its effectiveness in identifying heart-specific peptides.

## Discussion

The primary goal of our study is identifying peptides that target the ischemically damaged LV region. We performed phage display experiments in the ischemia–reperfusion mouse model and used three bioinformatic methods to analyze the data and nominate the hits for the validation in vitro. Three peptides AZ#6, AZ#10, AZ#12 identified by SPEC demonstrated specific uptake by human induced pluripotent stem cell (iPSC)-derived cardiomyocytes and immortalized mouse cardiomyocytes HL1. Two of them AZ#6, AZ#10 demonstrated affinity towards hypoxia and ischemia-stimulated cardiomyocytes as well.

It is interesting that we were unable to confirm cardiomyocyte binding for any of the hits identified through the established and routinely-applied bioinformatic approaches, such as TOPIC and TOPIC20. We believe that this may be attributed to the high sequence similarity observed among the peptides in our samples. Notably, we observe a significant number of peptides binding the liver and kidney to be closely related to those bind the damaged LV region during the last round of the phage display. Consequently, we may have mistakenly considered peptides binding the damaged LV region as specific for the damaged region because identical peptides are not found in the kidney or liver. However, it is possible that highly similar peptides are present among peptides binding the kidney or liver, leading to potential false positives. The SPEC approach involves tracking the distribution of clusters containing similar peptides. It allows us to better account for the presence of closely related peptides and their distribution across different tissues. Notably, the peptides identified through SPEC exhibit improved performance in the subsequent in vitro validation experiments (Fig. 4C). By considering the distribution patterns of clusters with similar peptides, we are able to better discern the true specificity of the peptides for the target tissue. These results highlight the importance of addressing the challenges posed by sequence similarity in phage display data analysis. The incorporation of cluster analysis in SPEC proves instrumental in mitigating potential false positives and shortlists better performing peptides during in vitro validation.

The obtained hits were additionally evaluated for their enrichment across the phage display rounds. Interestingly, those clusters that were enriched over the phage display rounds in heart and ischemic region but had reducing number of counts in the kidney and liver do not validate as binders to cardiomyocytes during our in vitro experiments. This finding suggests that searching for enrichment patterns over the phage display rounds may be too stringent for effective data analysis and eliminates true-positive hits. Several factors could contribute to this outcome. One possibility is the involvement of technical aspects, such as sample loss during phage amplification steps or difficulties in retrieving phages from the tissues^15,16^. This makes a great limitation to the applied enrichment analysis as normalisation cannot account for the missing values. Another potential factor is library amplification bias, which favours clones that propagate faster^13,14^. To address these challenges and improve the quantifiability of the assay, designing phage libraries with unique phage identifiers incorporated into the phage DNA and specific to each individual phage in the library may be helpful. This approach would enable a more reliable and quantifiable analysis by facilitating accurate phage quantification throughout the experimental process.

Interestingly TriCo-20 library had very less clusters and more enriched according to read counts in the round 3 than PhD-12 library. Nevertheless damaged region-specific clusters were found only in PhD-12 library. This suggests that broader peptides representation is the last round of phage display support identification of specific hits.

It is important to note that the specificity of binging to the ischemically damaged LV region in this study does not imply exclusive binding to cells within that region. The selection of the damaged LV region in our study is based on visual examination of the heart. We rely on the distinctive morphological differences observed in damaged tissue to identify the ischemically damaged region. However, it is important to acknowledge that there are viable heart cells in the damaged region. Therefore, we included two peptides (AZ#6 and AZ#7) that bind both the remote heart and the damaged LV region and one peptide that binds to remote heart (AZ#12) identified in SPEC for further characterization, acknowledging potential overlap and allowing for a comprehensive assessment of their targeting abilities. All of them demonstrated binding to human iPSC-derived cardiomyocytes in “healthy” conditions in vitro.

Among the strongest binders to iPSC-derived cardiomyocytes, AZ#6 and AZ#10 demonstrated affinity towards hypoxia and ischemia-stimulated cardiomyocytes as well. In contrast, AZ#12 did not show binding or uptake in these conditions. This is a strong indication that AZ#12 has specificity towards “healthy” cardiomyocytes as expected based on the in vivo phage display data analyzed by SPEC.

An important consideration for interpreting the in vivo results is the composition of cardiac tissues. Although cardiomyocytes constitute the majority of cells in the heart, there are other cell types, such as fibroblasts, endothelial cells, smooth muscle cells, and immune cells^23^. During myocardial infarction, immune cells, in particular, are enriched in the ischemic region^24,25^. Given this cellular heterogeneity, it is possible that some peptides identified through our in vivo phage display screen may bind to cell types other than cardiomyocytes. Consequently, these peptides may not exhibit binding specifically to cardiomyocytes in our follow-up in vitro assays. It is challenging to definitively rule out this possibility, especially considering the clinical potential of cardiac targeting peptides, which led us to focus primarily on the interaction of peptides with cardiomyocytes.

CTPs can potentially be utilized in various applications. One prominent application is that these peptides can serve as vehicles for delivering therapeutic agents specifically to the damaged myocardium by conjugating with these agents^26–28^. This approach could minimize off-target effects of therapeutic agents and enhancing their therapeutic efficacy. This potential of CTPs can be extended to regenerative medicine by facilitating the targeted delivery of stem cells, growth factors, or other regenerative agents to the site of myocardial injury^29^. This targeted regenerative therapy holds promise for promoting tissue repair and cardiac regeneration, offering new possibilities to improve outcomes of HF patients. The application of CTPs in diagnostic and imaging techniques represents another promising avenue of exploration^30^. The specific binding of CTPs to cardiac tissue can enable accurate detection and monitoring of MI and other cardiac pathologies, these peptides can serve as molecular probes for non-invasive imaging modalities, such as positron emission tomography and magnetic resonance imaging^31^. The use of CTPs therefore holds great potential for improving the diagnosis, treatment, and overall outcomes of patients with myocardial infarction.

In summary, the combination of phage display technology, NGS, and advanced bioinformatic approaches significantly enhances our capabilities in identifying peptide ligands specific for cardiac targets.

## Methods

### Cardiac ischemia and reperfusion injury in mice

The study involving animal experimentation was conducted following the guidelines set by the National Institute of Health (NIH) for the use of experimental animals. The study protocol was approved by the Animal Ethics Committee at Gothenburg University (Gothenburg Ethical Review Board number Ea (1173-2017)). The study complies with the ARRIVE guidelines. Male C57BL/6 mice, aged 10–12 weeks and weighing approximately 25 g, were obtained from Charles River and housed under controlled conditions. The mice were kept on a 12 h light/12 h dark cycle, at an ambient temperature of 21–22 °C, and 50% humidity.

To induce transient occlusion of the left anterior descending coronary artery (LAD), mice were anesthetized using a mixture of 2–3% Isoflurane (Forene) and oxygen, intubated and connected to a ventilator (MiniVent Ventilator for Mice, Model 845, Harvard Apparatus). The mice were ventilated with air (approximately 800 mL/min) and oxygen (approximately 100 mL/min) at a rate of approximately 230 strokes per minute. Core body temperature was continuously monitored and maintained at 35–36.5 °C using a heating operating table and heating lamp.

Electrodes were inserted under the skin to register and monitor the electrocardiogram (ECG) using a PharmLab system (Paris, France). A left thoracotomy was performed at the fourth intercostal space, approximately 2 to 3 mm to the left of the sternum, with the aid of a rib spreader to keep the incision open. The pericardium was opened, and the LAD originating from the left ventricle was identified. Temporary occlusion of the LAD was achieved using an 8–0 suture (Braun, Kronberg im Taunus). After 55 min of LAD occlusion, the suture was removed to allow reperfusion of the myocardial tissue. The chest was then sutured, and the mice were closely monitored while maintaining body temperature and ventilation until they regained consciousness and could be disconnected from the equipment.

### Electrocardiography

ECG recordings were acquired during MI surgery using tree-lead subdermal needle electrodes, connected to a PowerLab 8/30 data acquisition device (model ML870, ADInstruments) and an animal Bio Amp biological potential amplifier (model ML136, ADInstruments) as previously reported^32^. RR-, PR-, QRS- and QT- intervals, P-duration, P-, Q-, R- S- and T-amplitudes, ST-height and heart rate were analyzed using the ECG Analysis module in the Lab-Chart Pro.

### Echocardiography

Echocardiography to assess heart function was performed 3 days after cardiac LAD ischemia–reperfusion injury^33^. Mice were anesthetized using 2.5% isoflurane (Forene) mixed with air and then maintained on 1.5–2% isoflurane during the assessment. During the examination, the mice were kept on a Physio Plate (Visualsonics) to keep normal body temperature and to monitor ECG and respiration. The ultrasound probe (MS400, 18–38 MHz) was connected to an ultrasound biomicroscope (Vevo 2100 System, Visualsonics). ALV long-axis parasternal B-mode view was captured, followed by a 90° clockwise rotation of the ultrasound probe adjusted to obtain short axis B-mode views at the following three levels: (i) at level of LV base, (ii) at an intermediate position, approximately at the level of the papillary muscles, and (iii) at the apex level. From these acquisitions, a 3D reconstruction of the LV geometry and the calculation of diastolic and systolic LV volumes (LVEDV and LVESV), EF was done using the modified Simpson’s method (Vevo Lab 5.6.1, VisualSonics)^34^.

All measurements were obtained by averaging three consecutive cardiac cycles, with the treatment group of animals blinded to the sonographer.

### In vivo phage display

Two different phage libraries encoding 12 amino acid peptides (PhD-12, New England Biolabs) and 20 amino acid peptides (TriCo-20, Creative Biolabs) were used in these experiments. TriCo-20 has a sequence diversity of 1.1 × 10^10^ and consisted of 20-amino acid peptides linked to pIII of M13 bacteriophage via a serine linker. PhD-12 has a titer of 2 × 10^13^ and consisted of 12-amino acid peptides connected to pIII of M13 bacteriophage via a (glycine)_3_-serin linker. Four days after ischemia–reperfusion injury surgery, each phage library was administered intravenously via tail vein into two anesthetized animals. Animals were perfused through the right ventricular with at least 10 ml HBSS under anesthesia 15 min after injection to minimize non-specific binding of phages. Thereafter, ischemic LV, remote heart (the rest of the heart after removing ischemic LV and without aorta), kidney and liver were dissected and collected in PBS. The infarcted area was identified by discoloration, excised and all tissues were homogenized with a QIAGEN TissueLyser II and incubated with dispase II in RPMI medium for 40 min at 37 °C. Addition of RPMI medium (#11875093, Gibco) containing 10% fetal bovine serum (Gibco) stopped digestion and cell suspension was then transferred through a 100 µm mesh, followed by 30 µm mesh to exclude cell clumps.

### Phage purification, recovery and preparation for next selection round

Phage purification from the tissue homogenates was achieved by precipitation with 200 µl PEG/NaCl to 800 µl of homogenized tissue on ice for 1 h. Subsequently, phages were pelleted by centrifugation at 12,000×*g* for 15 min at 4 °C, the pellet was dissolved in 25 µl PBS and added to a log phase culture of ER2738 *E. coli* in LB medium containing tetracycline. Bacteria/phage infection was incubated for 30 min shaking at 225 rpm, before LB medium containing tetracycline and glucose was added for incubation overnight. Bacteria were removed by centrifuging twice at 4600 rpm for 15 min at 4 °C and the bacteria pellet was used to prepare a glycerol stock of the panning round by adding LB medium containing tetracycline and 15% glycerol. Glycerol stocks were stored at − 80 °C. Phage precipitation from LB medium with PEG/NaCl (5:1 ratio between LB medium and PEG/NaCl) on ice for 1 h was followed by centrifugation at 4600 rpm for 15 min at 4 °C to pellet phages. Phages were resuspended in sterile PBS and phage titer was determined by measuring absorption at 269 and 320 nm on Nanodrop (Thermo Fisher Scientific, Waltham, MA, USA) and calculated as followed: virions/ml = (A269-A320)*6*10^16^/number of bases per virion^35,36^.

### Sample preparation and next generation sequencing

Bacteria, infected with phage, were grown in tetracycline containing LB medium at 37 °C with shaking at 225 rpm. QIAprep Spin Miniprep Kit was used to purify phage plasmid dsDNA. Phage inserts were amplified by PCR by mixing of 20 ng purified DNA, 0.5 µM of a primer pair (forward: 5’-TTGTCGTCTTTCCAGACGTT-3’; reverse: 5’-GCAAGCTGATAAACCGATACA-3’) covering the peptide inserts in pIII,^18^ Phusion Flash High Fidelity PCR Master Mix 1 × and nuclease free water in a final volume of 10 µl. The following PCR cycling conditions were used: 98 °C for 2 min, then for 25 cycles 96 °C for 5 s, 64 °C for 6 s, 72 °C for 5 s and as a last step 1 min at 72 °C. Agencourt AmPure XP purification system was used to purify the amplicons and high sample purity and concentration determination was verified on a 5200 Fragment Analyzer system (Agilent Technologies) using the dsDNA 915 Reagent kit (35-5000 bp). Amplicons were then sequenced by NGS. Input DNA concentration was adjusted to 0.067 ng/µl using QuBit dsDNA assay. Samples were run with standard paired end 2 × 150 bp read sequencing on an Illumina NextSeq500 machine. Using the Burrows Wheeler aligner (BWA)^37^, samples were mapped on the pIII coat protein of the M13 bacteriophage and quality controlled using the phred score. The fastq reads were then scanned for the expected flanking sequences of interest (CCTTTCTATTCTCAC-GCCGAAACTGTTGAA), allowing for 2 mismatches per flank. The DNA sequences within the flanking regions were extracted, counted, and translated into protein for further analysis.

### Data analysis

All filtration steps were performed in Rstudio. Firstly, sequences containing stop codons or not amino acid residues (NA) were eliminated. Next, the remaining sequences were checked for conformity to the library length and pattern (S-peptide sequence-GGGS for PhD-12 library and S-peptide sequence-S for TriCo-20), ensuring the presence of the correct amino acids preceding the targeting peptides and to the appropriate linker sequence. Finally, any duplicated sequences were removed from the dataset, ensuring a unique representation of the identified peptides.

Multiple sequence alignment was done with the help of R-package MSA^38^ freely available on the Bioconductor platform^39^.

Clustering of the peptide sequences was performed utilizing R-package DECIPHER^40^ and the subsequent libraries “DistanceMatrix” and “IdClusters” also available on the Bioconductor platform^39^. The clustering was achieved with the weighted hierarchical clustering method WPGMA and the maximum edge length separating the sequences in the same cluster ranged from 0.3 to 0.7, depending on the library sequence length. Clustering of the peptides at the low scale (TOPIC) was run locally, clustering of all peptides in the library was performed on a remote server to have enough computational power. The enrichment analysis was carried out with the help of the R-package ComplexHeatmap from BioConductor^39^. The data was firstly normalized through log2 to account for the wide range of sequence reads throughout the rounds. Due to the presence of N/A values of sequences that were not read through all rounds, they were converted to the value of “0”.

### Peptide synthesis

Peptides were synthesized by GenScript Biotech with a Lysine-attached FITC reporter. Lyophilised peptides were dissolved in water or 20–55% DMSO based on peptide solubility.

### Cell culture

iCell cardiomyocytes (#01434, Fujifilm Cellular Dynamics) were thawed according to manufacturer’s instructions in plating medium (Fujifilm Cellular Dynamics) and seeded in fibronectin-coated (Sigma-Aldrich) 384-well Cell Carrier Ultra plates (#6007558, PerkinElmer) at a density of 7 × 10^5^ cells/well. Plates were incubated 30 min at RT followed by incubation at 37 °C with 5% CO_2_ and 95% relative humidity. Next day plating medium was switched to maintenance medium (Fujifilm Cellular Dynamics) and plates were incubated 72 h at 37 °C before treatment with peptides.

HL-1 cells were cultured in Claycomb medium (#51800C, Merck Millipore), with 0.1 mM Norepinephrine (#A-0937, Merck Millipore), 2 mM L-Glutamine and 10% fetal bovine serum (FBS HL-1 cell screened, #TMS-016-B, Merck Millipore). Norepinephrine stock (10 mM) was prepared in 30 mM L-Ascorbic acid, sodium salt (#A-7631, Merck Millipore). Cells were seeded in fibronectin-coated (Sigma-Aldrich) 384-well Cell Carrier Ultra plates (#6007558, PerkinElmer) at a density of 5 × 10^3^ cells/well and incubated 72 h with 5% CO_2_ and 95% relative humidity before treatment with peptides.

HepG2 cells were cultured in high glucose DMEM (#11965118, Thermo Fischer Scientific) with 2 mM glutamine, 0.1 mM MEM Non-Essential Amino Acids, and 10% fetal bovine serum (Gibco) at 37 °C in 5% CO_2_ and 95% relative humidity. Cells were plated in 384-well Cell Carrier Ultra plates (#6007558, PerkinElmer) at a density of 3.5 × 10^3^ cells/well and incubated 72 h before treatment with peptides.

### Hypoxia stimulation of cardiomyocytes in vitro

iCell cardiomyocytes were loaded into pre-warmed (37 °C) incubator and hypoxia was initiated using (0.8% O_2_ and 5% CO_2_) hypoxic gas. After 24 h hypoxia-reoxygenation experiments were performed. The cells were moved to normoxic condition (19% O_2_ and 5% CO_2_) and incubated for 6 hours^22^.

### Ischemia stimulation of cardiomyocytes in vitro

iCell cardiomyocytes media was changed to nutrition-depleted media (DMEM with no glucose, #11966-025, Thermo Fischer Scientific), 1% NEAA (#11140-035, Gibco) and 0.1% Penicillin/Streptomycin solution (Thermo Fischer Scientific) and cells were loaded into pre-warmed (37 °C) incubator and hypoxia was initiated using (0.8% O_2_ and 5% CO_2_) hypoxic gas. After 24 h media was changed to maintenance medium (Fujifilm Cellular Dynamics) and hypoxia-reoxygenation was performed. The cells were moved to normoxic condition (19% O_2_ and 5% CO_2_) and incubated for 6 hours^22^.

### Live cell imaging

Prior to assay, cells were treated with candidate peptides at concentration of 10 µM. Control was treated with DMSO. Cells were incubated with peptides for 1 h at 37 °C. This was followed by 2 washes and exchange to DMEM/F12 Phenol Red-free Medium (Thermo Fischer Scientific) containing Hoescht 33342 dye (#62249, Thermo Fischer Scientific). After 10 min samples were live imaged on Cell Voyager 7000S or 8000 microscope (Yokogawa) at 37 °C with 40 × oil or 20 × and 60 × water objectives applying the same imaging settings to all the conditions (Exposure time 250 ms, Laser power 30%, Binning 2 × 2 for all the channels). For the image visualization, the same brightness/intensity settings were used for all the samples within the same experiment.

### Fixed cells imaging

After the live cell imaging, iPSC-derived cardiomyocytes were fixed by incubating them for 20 min in 4% paraformaldehyde (PFA). After fixation, the PFA was washed away using PBS (-Ca/-Mg) and Cell mask deep red plasma membrane dye (#C10046, TheromFisher Scientific) and Hoechst 33342 (#62249, ThermoFisher Scientific) were added for 15 min followed by three washes with PBS. Afterwards cells were imaged on Cell Voyager 7000S or 8000 microscope (Yokogawa) at room temperature applying the same imaging settings to all the conditions.

For troponin T visualization and any background protein signal was blocked by incubating the cells for 1 h at 37 °C in a blocking solution (PBS -Ca/-Mg containing 10% FBS and 1% Triton X-100 Surfact-Amps Detergent Solution (#85111, ThermoFisher Scientific). The blocking solution was replaced with a solution of PBS -Ca/-Mg containing 5% FBS and a 1:1000 dilution of primary antibody against cardiac troponin T (#MA5-12960, ThermoFisher Scientific), and the cells were incubated overnight. The following day, the primary antibody was washed off four times with PBS -Ca/-Mg, and the cells were then incubated for 10 min with PBS -Ca/-Mg containing 20 µM Hoechst 33342 (#62249, ThermoFisher Scientific) for nuclei staining. After 10 min, the Hoechst 33342 was removed by washing the cells three times with PBS -Ca/-Mg. Finally, images were acquired at a magnification of 4X using the ImageXpress Micro Confocal High-Content Imaging System (Molecular Devices).

### Figures preparation

Biorender was used for the generation of panels A, B and D on Fig. 1.

### Generative AI and AI-assisted technologies in the writing process

During the preparation of this work ChatGPT 3 was used for spelling and grammar check. The authors reviewed and edited the content as needed.

### Supplementary Information

Supplementary Figures.

## Data Availability

All data needed to evaluate the conclusions in the paper are present in the paper and/or the Supplementing Information. Additional data related to this paper and R scripts may be requested from the corresponding authors.
