# Supplementary material for: In vivo phage display identifies novel peptides for cardiac targeting
Source: Sci Rep. 2024 May 28;14:12177. doi: 10.1038/s41598-024-62953-9 (PMC11133476; doi:10.1038/s41598-024-62953-9)
Supplement: Supplementary file 1 — Supplementary Figures. [file 41598_2024_62953_MOESM1_ESM.docx]

**
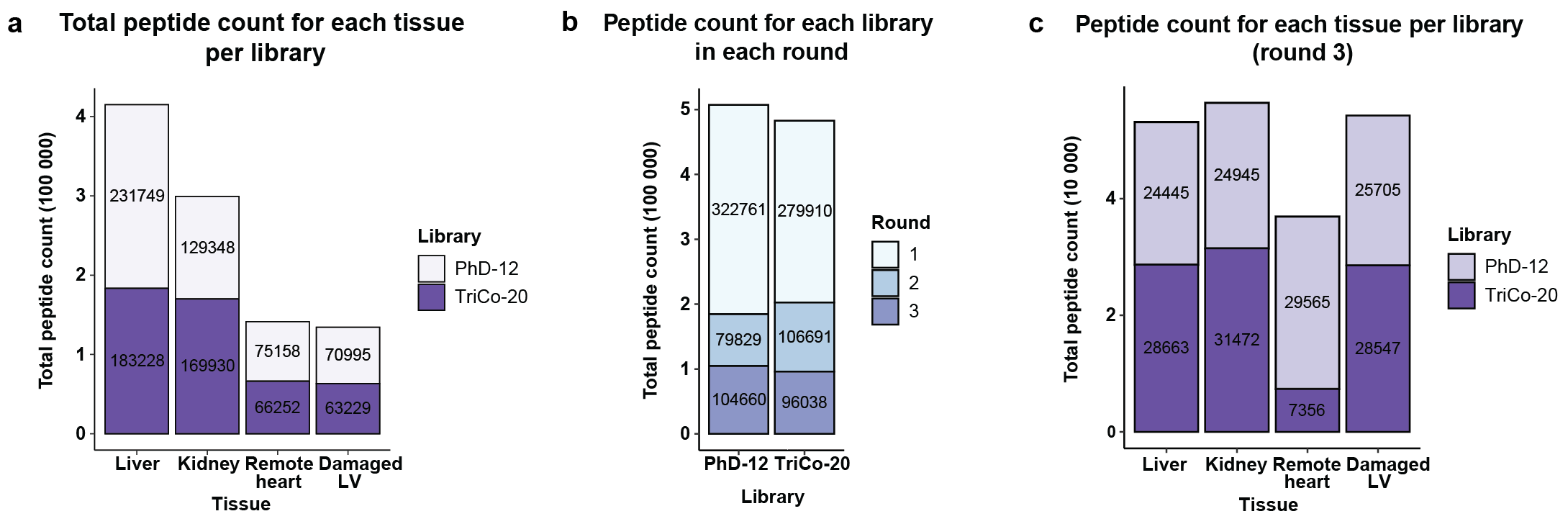
Supplementary materials**

**Figure S1. Libraries representation in different tissues and rounds.**

(a) Library representation across different tissues summed across three rounds.

(b) Libraries representation across different rounds of the phage display across all the investigated tissues.

(c) Filtered data set was used to calculate the number of reads in each tissue in the final round of the phage display experiment per library.


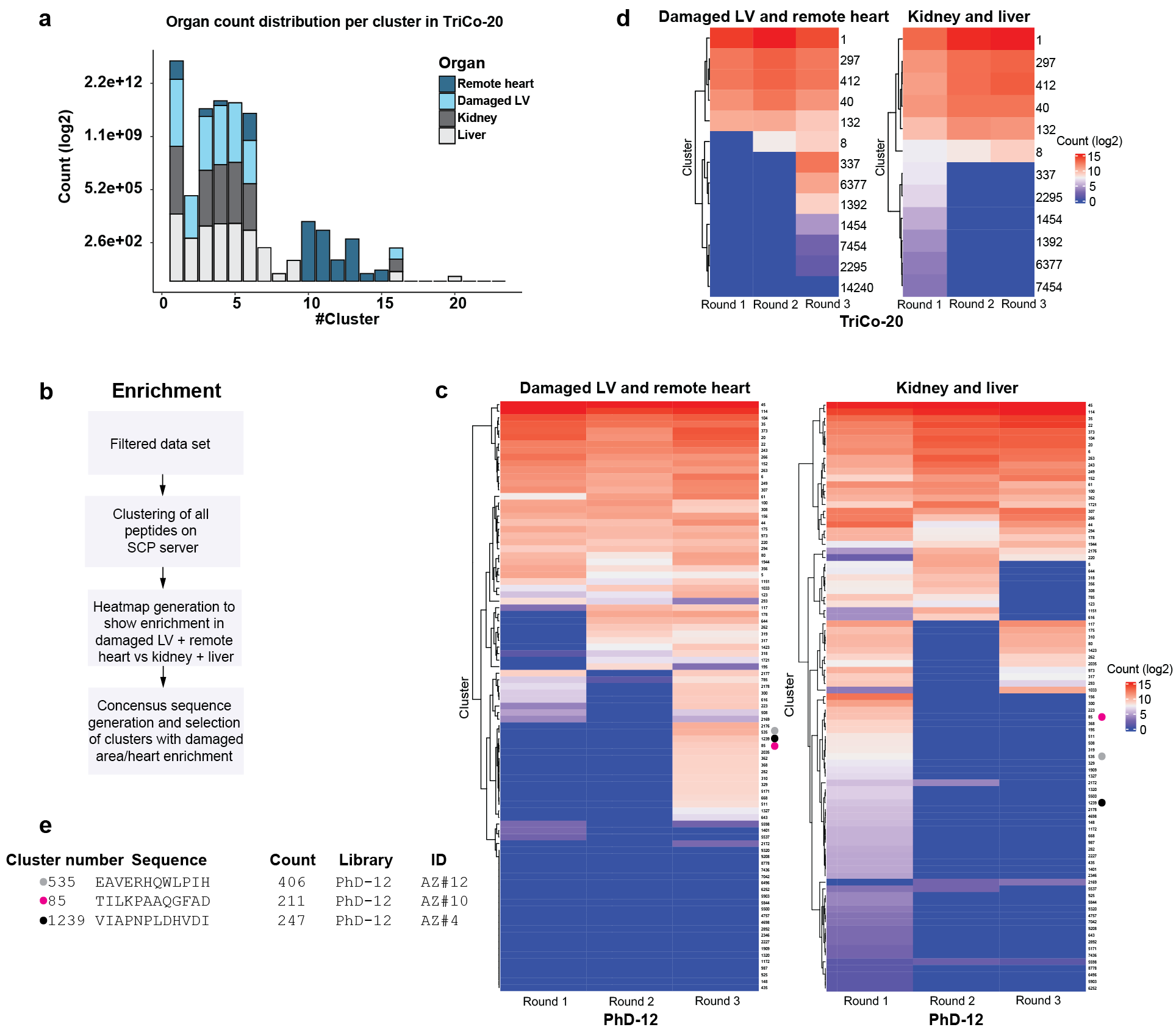


**Figure S2. Representation of clusters identified in SPEC and their enrichment across the rounds.**

(a) Visual representation of clusters identified in panning round 3 across different tissues for TriCo-20 library.

(b) Description of the Enrichment data analysis. All the peptides identified in all three rounds of the phage display were clustered on the SPC server. Heatmap is generated to illustrate clusters’ performance across the panning rounds.

(c) Heatmaps representing PhD-12 library peptide counts in identified clusters across three rounds. Red colour represents clusters with the highest counts, and blue corresponds to 0 counts. Magenta, grey and black circles highlight clusters identified as hits.

(d) Consensus peptide sequences highlighted as hits in C.

(e) Heatmaps representing peptide counts in identified clusters across all panning rounds for TriCo-20 library.

**
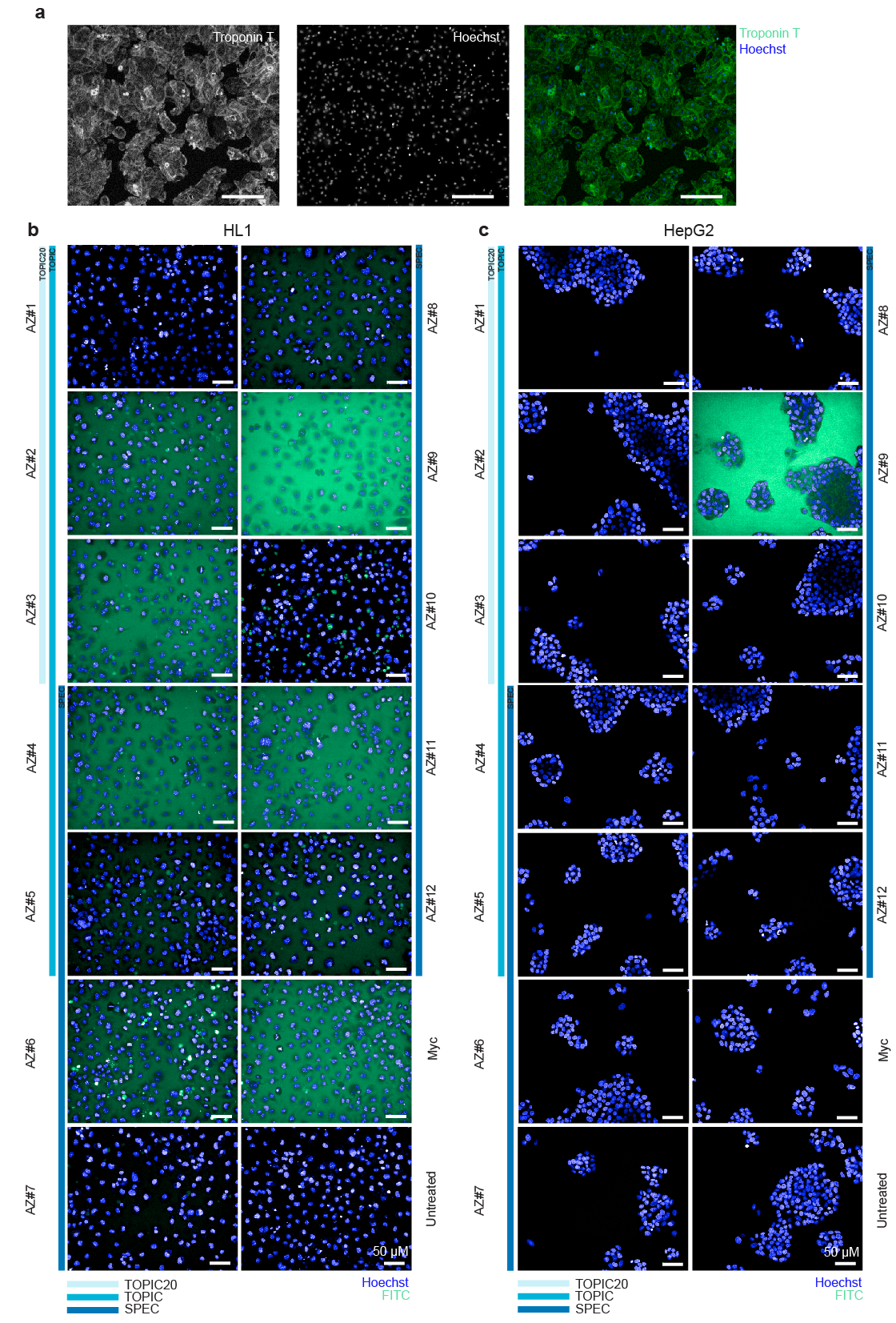
**

**Figure S3. *In vitro* validation of identified peptides with HL1 and HepG2 cells.**

(a) Troponin T staining of the iPSC-derived cardiomyocytes. Scale bar = 100 μm.

(b), (c) HL1 (a) and HepG2 (b) cells were incubated with the indicated peptides labeled with the FITC fluorophore for 1 hour. Afterward, peptides were washed away by changing the media, and live cell imaging was performed using high throughput confocal microscope. Blue lines indicate the approach used to identify the indicated peptide as a hit. Scale bar = 50 μm.

**
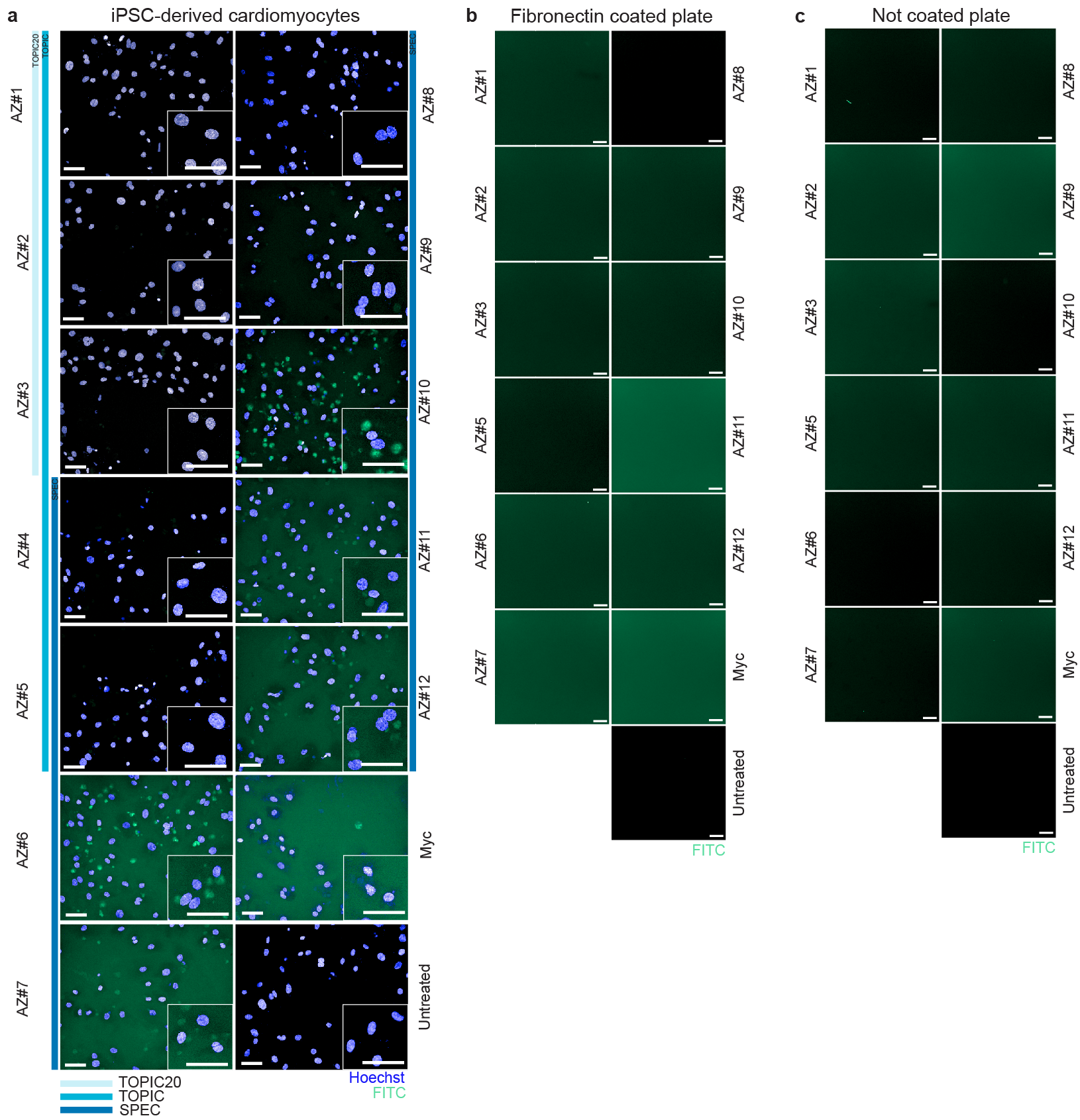
**

**Figure S4. Images from *in vitro* validation of identified peptides with human iPS-derived cardiomyocytes.**

(a) Representative confocal fluorescence microscopy images of live human iPSC-derived cardiomyocytes incubated for 1 hour with indicated peptides labeled with the FITC fluorophore. Hoechst33342 dye was used for nuclei visualization. Blue lines indicate approaches used to identify the indicated peptide as a hit. Scale bar = 50 μm.

(b) Representative confocal fluorescence microscopy images of the plate surface coated with fibronectin and incubated for 1 hour with indicated peptides labeled with the FITC fluorophore. Scale bar = 50 μm.

(c) Representative confocal fluorescence microscopy images of the plate surface without fibronectin coating and incubated for 1 hour with indicated peptides labeled with the FITC fluorophore. Scale bar = 50 μm.


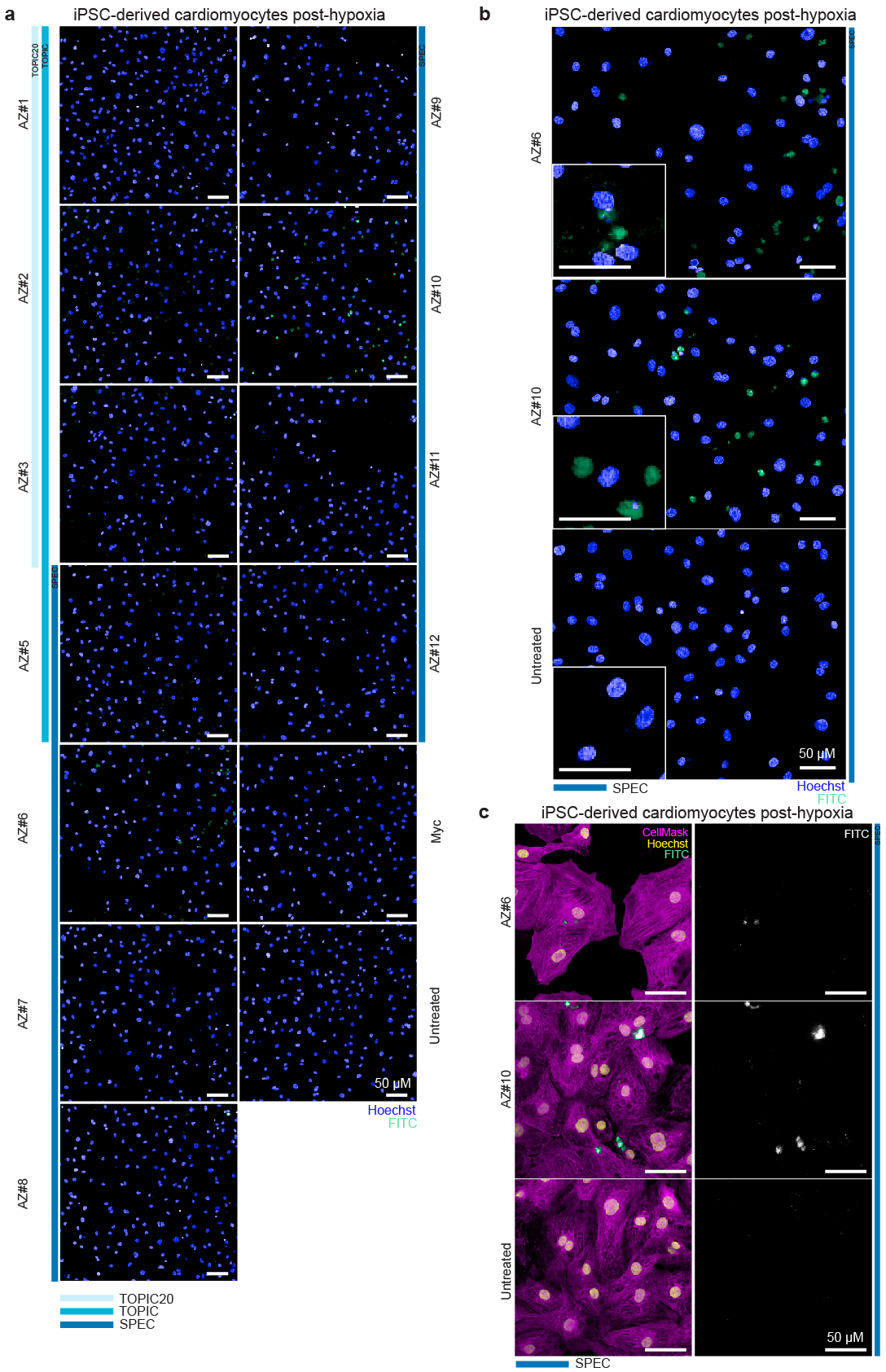


**Figure S5. Images from *in vitro* validation of identified peptides with human iPS-derived cardiomyocytes stimulated with hypoxia.**

(a) IPSC-derived cardiomyocytes were incubated for 24 hours in hypoxia (0.8% O_2_) condition followed by 6 hours of reoxidation. Afterward, cells were incubated with the indicated peptides labeled with the FITC fluorophore for 1 hour. Peptides were washed away by changing the media, and live cell imaging was performed using high high-throughput confocal microscope. Blue lines indicate the approach used to identify the indicated peptide as a hit. Scale bar = 50 μm.

(b) Enlarged images for the indicated peptides from panel (a). Scale bar = 50 μm.

(c) Human iPSC-derived cardiomyocytes from (a) were fixed after 1 hour of incubation with FITC-labelled peptides. Cell mask and Hoechst33342 dyes were used for membrane and nuclei visualization, respectively. Scale bar = 50 μm.


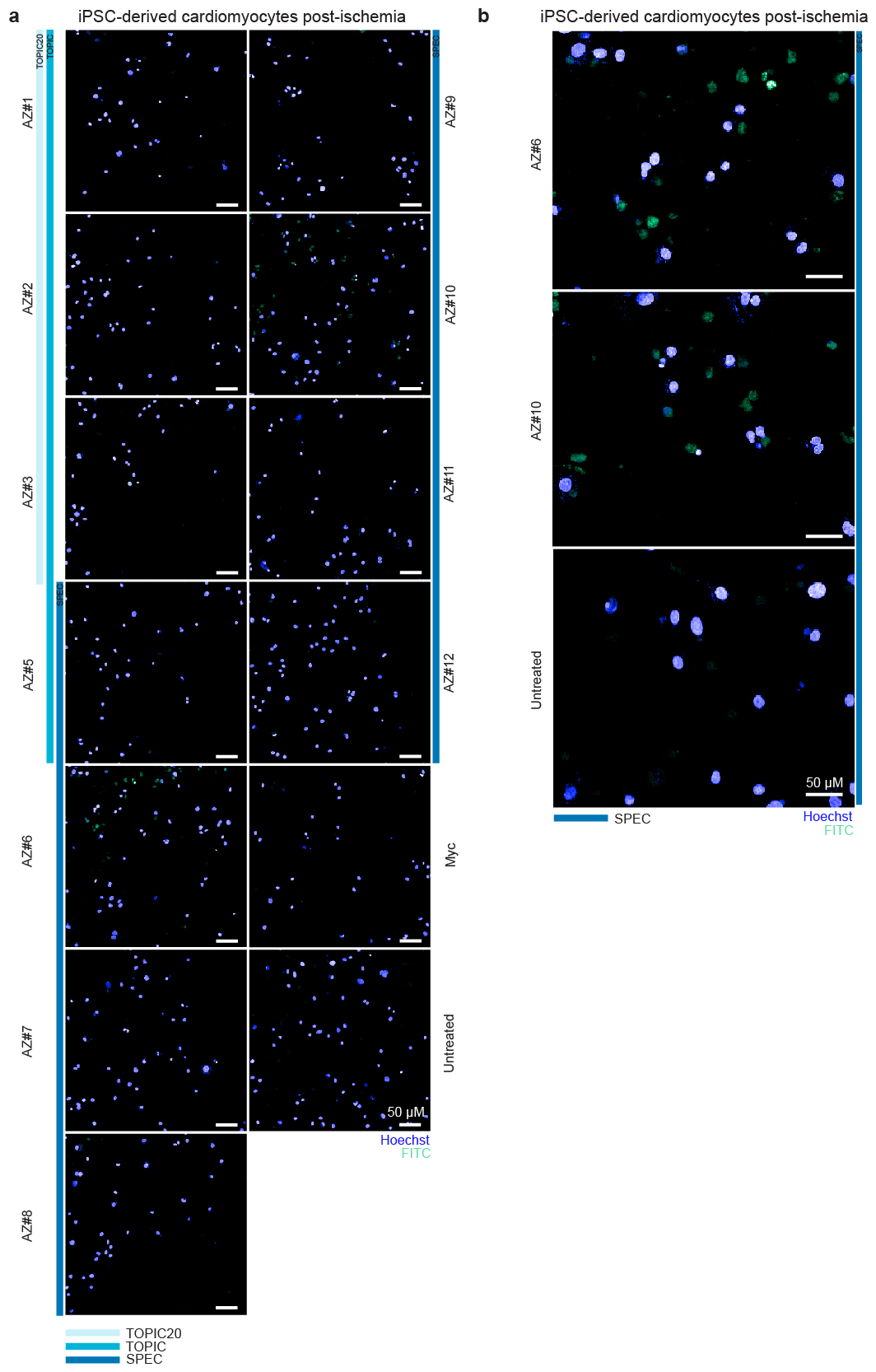


**Figure S6. Images from *in vitro* validation of identified peptides with human iPS-derived cardiomyocytes stimulated with ischemia.**

(a) IPSC-derived cardiomyocytes were incubated for 24 hours in hypoxia (0.8% O_2_) with nutrition depletion followed by 6 hours of reoxidation and recovery in the full media. Afterward, cells were incubated with the indicated peptides labeled with the FITC fluorophore for 1 hour. Peptides were washed away by changing the media, and live cell imaging was performed using high high-throughput confocal microscope. Blue lines indicate the approach used to identify the indicated peptide as a hit. Scale bar = 50 μm.

(b) Enlarged images for the indicated peptides from panel (a). Scale bar = 50 μm.
